# Inhibition of the Myotoxicity Induced by *Bothrops jararacussu* Venom and Isolated Phospholipases A_2_ by Specific Camelid Single-Domain Antibody Fragments

**DOI:** 10.1371/journal.pone.0151363

**Published:** 2016-03-30

**Authors:** Nidiane D. R. Prado, Soraya S. Pereira, Michele P. da Silva, Michelle S. S. Morais, Anderson M. Kayano, Leandro S. Moreira-Dill, Marcos B. Luiz, Fernando B. Zanchi, André L. Fuly, Maribel E. F. Huacca, Cleberson F. Fernandes, Leonardo A. Calderon, Juliana P. Zuliani, Luiz H. Pereira da Silva, Andreimar M. Soares, Rodrigo G. Stabeli, Carla F. C. Fernandes

**Affiliations:** 1 Fundação Oswaldo Cruz, Fiocruz Rondônia, Porto Velho-RO, Brazil; 2 Universidade Federal Fluminense, UFF, Rio de Janeiro-RJ, Brazil; 3 Universidade Federal de Rondônia, UNIR, Porto Velho-RO, Brazil; 4 Empresa Brasileira de Pesquisa Agropecuária, Embrapa, Porto Velho-RO, Brazil; 5 Centro de Pesquisa em Medicina Tropical, CEPEM, Porto Velho-RO, Brazil; Universidad de Costa Rica, COSTA RICA

## Abstract

Antivenoms, produced using animal hyperimmune plasma, remains the standard therapy for snakebites. Although effective against systemic damages, conventional antivenoms have limited efficacy against local tissue damage. Additionally, the hypersensitivity reactions, often elicited by antivenoms, the high costs for animal maintenance, the difficulty of producing homogeneous lots, and the instability of biological products instigate the search for innovative products for antivenom therapy. In this study, camelid antibody fragments (VHH) with specificity to Bothropstoxin I and II (BthTX-I and BthTX-II), two myotoxic phospholipases from *Bothrops jararacussu* venom, were selected from an immune VHH phage display library. After biopanning, 28 and 6 clones recognized BthTX-I and BthTX-II by ELISA, respectively. Complementarity determining regions (CDRs) and immunoglobulin frameworks (FRs) of 13 VHH-deduced amino acid sequences were identified, as well as the camelid hallmark amino acid substitutions in FR2. Three VHH clones (KF498607, KF498608, and KC329718) were capable of recognizing BthTX-I by Western blot and showed affinity constants in the nanomolar range against both toxins. VHHs inhibited the BthTX-II phospholipase A_2_ activity, and when tested for cross-reactivity, presented specificity to the *Bothrops* genus in ELISA. Furthermore, two clones (KC329718 and KF498607) neutralized the myotoxic effects induced by *B*. *jararacussu* venom, BthTX-I, BthTX-II, and by a myotoxin from *Bothrops brazili* venom (MTX-I) in mice. Molecular docking revealed that VHH CDRs are expected to bind the C-terminal of both toxins, essential for myotoxic activity, and to epitopes in the BthTX-II enzymatic cleft. Identified VHHs could be a biotechnological tool to improve the treatment for snake envenomation, an important and neglected world public health problem.

## Introduction

Snakebites represent a relevant public health problem, especially in subtropical and tropical countries. Affecting mainly the rural population, about 5 million snakebites occur each year worldwide, causing approximately 100,000 deaths [1–3]. A large number of the victims experience permanent physical sequelaes due to inflammatory, hemorrhagic, coagulant, neurotoxic and myotoxic effects occasioned by the venom components. These signs are often aggravated by the difficulty of accessing health services in less developed regions [2,4].

It has been described that, worldwide, there are four families of venomous snakes, i.e. Viperidae, Elapidae, Atractaspididae, and Colubridae [5]. Viperidae and Elapidae families cause the most venomous bites. Beloging to the Viperidae family, the *Bothrops* genus is responsible for the majority of snake envenoming in Central and South America, occasioning both high morbidity and mortality [6].

Diagnosis of snakebite is based on clinic-epidemiological evalution and the current treatment consists, besides supportive care, of the intravenous administration of antivenoms [7]. To manufacture antivenom, hyperimmune plasma is obtained from venom-immunized horses or sheep and subjected to physical and chemical processes to generate monovalent or polyvalent serum, containing polyclonal immunoglobulins G (150 kDa) or antibody fragments, such as F(ab’)_2_ (100 kDa) or Fab (50 kDa), which are not affinity purified [8].

Although they are effective against systemic toxic effects, conventional antivenoms poorly neutralize venom toxins in deep tissues, due to the discrepancies between the pharmacokinetic profiles of the low molecular weight toxins and antivenoms [9]. Additionally, the hypersensitivity reactions, often elicited by heterologous serum, the high costs for animal maintenance, the difficulty of producing homogeneous lots, the instability of biological products, together with animal rights and welfare instigate the search for innovative products for snakebite therapy [8].

Among proposed innovations the use of monoclonal antibodies and recombinant antibody fragments, like single chain variable fragment (scFv), stand out [10–12]. While the high immunogenicity, as well as the high cost for monoclonal antibodies production limit the use of that technology, scFvs usually show lower affinity when compared with the antigen recognition region of conventional antibodies [13].

To overcome these issues, studies have proposed the use of recombinant antigen binding domains derived from camelid heavy chain antibodies, called VHH, for antivenom production [14]. With about 15 kDa, one-tenth the size of whole antibodies, VHH possess attractive physico-chemistry, pharmacokinetic and pharmacodynamic properties, and shows low immunogenicity. Besides conserving the conventional antibodies’ benefits in terms of affinity and selectivity, they present high stability, solubility and lower production cost, when compared with human or murine antibodies [15]. VHHs present higher tissue penetration ability, and can act as potent enzyme inhibitors by penetrating in the toxin enzymatic clefts, normally inaccessible to conventional VHs [16,17]. Thus, VHHs have emerged as versatile biotechnological tools for antivenom development. However, antivenoms composed entirely of small antibody fragments, including VHHs, would have limited therapeutic efficacy because of their short serum half-life profiles. Therefore, different strategies have been explored in order to extend the serum half-life of VHHs [18]. Furthermore, the development of antivenoms based on a mixture of high (IgG; F(ab’)2) and low (Fab, scFv, VHH) molecular mass antibodies, could help match the pharmacokinetic profiles of venoms, improving antivenom biodistribution, stability, and toxin neutralization while reducing side effects in humans. Besides allowing for the neutralization of toxins by small fragments in tissue compartments, the formed toxin-VHH complex can be eliminated rapidly through renal excretion. In addition, these preparations ensure that a significant concentration of high molecular mass antibodies remains in circulation to neutralize toxins later in the course of envenomation [14].

For VHH production, two myotoxic phospholipases A_2_ (PLA_2_), obtained from *B*. *jararacussu* venom, were used as antigens. BthTX-I, a Lys-49 PLA_2_-like, without enzymatic activity, and BthTX-II, an enzymatically active Asp-49 PLA_2_. With 76% amino acid sequence similarity and a molecular mass of 14 kDa, both toxins correspond to 40–50% of the venom dry weight, and play an important role in muscle necrosis [19,20].

Evaluating the features of VHH, the characteristics of bothropstoxins, and the necessity for developing new approaches to support the treatment or even to diagnose snakebites, this study aimed to select VHHs capable of recognizing and neutralizing myotoxic effects triggered by BthTX-I, BthTX-II, and *B*. *jararacussu* venom.

## Materials and Methods

### Ethics statements

Experimental procedures involving animals were carried out in accordance with the recommendations of the National Council for the Control of Animal Experimentation (CONCEA), and were approved by the Ethics Commission on Animal Use (CEUA) of Fiocruz Rondônia under protocol 2012/11. Animal health status was checked routinely by a veterinary. In order to verify the study viability and to determine a protocol for experimental humane endpoints, a pilot study was conducted previously, following the CONCEA Normative Resolution No. 23, of July 23, 2015 (http://www.mct.gov.br/upd_blob/0237/237231.pdf). This protocol was conducted when severe neurotoxic and myotoxic signs were observed. All mice groups were monitored regularly (5 min intervals) until the end of experimental period and there were no unintended death of animals. After topical anesthesia (tetracaine ophthalmic drops) for retro-orbital bleeding, all animals were euthanized by Cervical dislocation, according to CONCEA Normative Resolution No. 13, of September 20, 2013 (http://www.mct.gov.br/upd_blob/0228/228451.pdf). The licenses related to access to Brasilian genetic resources for scientific purposes are: Instituto Brasileiro do Meio Ambiente e dos Recursos Naturais Renováveis–IBAMA, Instituto Chico Mendes de Conservação da Biodiversidade–ICMBio (Number: 27131–1) and Conselho de Gestão do Patrimônio Genético–CGEN/Brazil (Number 010627/2011-1).

### *Lama glama* immunization and immune response monitoring

One young adult male *L*. *glama*, with food and water *ad libitum*, was immunized at fortnightly intervals seven times with 100 μg of each BthTX-I and BthTX-II [19] via subcutaneous injections. Animal immune response was monitored by enzyme immunoassay. The assay was performed in triplicate. For this, microtiter plates (Nunc-MaxiSorp) were coated with 1 μg BthTX-I or BthTX-II in PBS/well and incubated overnight at 4°C. Wells were washed with PBS/ 0.05% Tween-20 (PBST) and unspecific sites blocked with blocking solution (BS – 3% of BSA plus 1% of skimmed milk in PBS) for 3 h. Subsequently, serial dilutions (1:10^2^; 1:10^3^;1:10^4^; 1:10^5^) of the serum, collected each week, were added to the wells and the plates were incubated for 12 h in BS at 4°C. After washing with PBST, rabbit anti-llama IgG_2_/IgG_3_ [21] at a 1:12000 dilution in BS was added. Excess antibody was removed by washing, and peroxidase conjugated mouse anti-rabbit IgG (Sigma Aldrich) was incubated at a 1:40000 dilution for 2 h in BS. TMB-Ultra (Millipore) was used to reveal the reaction. Absorbances were measured at 450 nm in a microplate reader (BioTek-Synergy HT). The negative control was performed using the llama pre-immune serum.

### VHH library construction

To construct the VHH immune library, blood was collected 3 days following the final boost, and lymphocytes were isolated with Ficoll-Paque Plus (GE Healthcare). Lymphocyte total RNA was extracted using Trizol Reagent (Invitrogen), and cDNA synthesis carried out with the SuperScript III First-Strand Synthesis System for RT-PCR (Invitrogen). Subsequently, PCR, performed using the cDNA as template and one pair of gene-specific primers, resulted in an amplification of the conventional and heavy-chain IgG repertoire gene fragments. Amplified gene fragments were electrophoresed and the purified heavy chain amplicon was used as template in a nested PCR, resulting in fragments consisting of about 400 bp [21]. VHH fragments were inserted into the **pHEN1-6xHis** phagemid vector, in frame with the M13 gene III for expression of VHH-6xHis-PIII fusion protein. Ligation products were transformed into electrocompetent *E*. *coli* TG1 (Stratagene) to generate the primary library of VHH. Library size was determined by plating the transformation product on 2YT agar plates with 100 μg/mL ampiclilin and 2% glucose (2YT/amp/glu). The attainment of recombinant M13KO7 phages (**New England Biolabs)** expressing anti-BthTX-I and anti-BthTX-II VHHs, as well as polyethylene glycol precipitation were performed as described by [22], and used for biopanning.

### Screening for anti-BthTX-I and anti-BthTX-II VHHs

BthTX-I and BthTX-II were used separately to select specific VHH-phages. One milligram of each toxin was immobilized on MaxiSorb immunotubes (Sigma-Aldrich) overnight at 4°C. Excess BthTX-I and BthTX-II were washed with PBST and the reactions were blocked in BS for 2 h. Phages expressing VHH repertory, previously incubated in BS, were added to the immunotubes and the samples were incubated at 37°C. After washing, phage elution was performed with 100 mM HCl and the reaction was neutralized with 1 M Tris-HCl. The eluants were transferred, separately, to *E*. *coli* TG1 (A_600_ 0.5), and the cultures were incubated at 37°C. After centrifugation, the supernatants were discarded, the pellet resuspended in 2YT, plated on 2YT/amp/glu, and incubated overnight at 30°C. VHH presence in the selected clones was checked by colony PCR and positive clones were selected to verify antigen binding specificity by ELISA. Thus, clones were expressed in 2YT/amp plus 1 mM IPTG for 16 h at 30°C. Following centrifugation, 50 μL of supernatants containing soluble VHHs were used to perform ELISA assay, as described under immune response monitoring. Llama BthTX-I and BthTX-II immune serum was used as positive controls. The negative control was carried out using the llama pre-immune serum. Positive clones were sequenced, analyzed and submitted to GenBank.

### Expression and Purification of anti-BthTX-I and anti-BthTX-II VHHs

To perform the expression of soluble VHHs, selected VHH-pHEN1-6xHis were electroporated into the non-supressor strain *E*. *coli* HB2151. Clones were grown in 2YT/amp (1 L) under shaking at 37°C (A_600_ = 0.5). To induce VHH expression, 1 mM IPTG was added to the reactions, and the cultures were grown for 16 h at 30°C. Samples were centrifuged, and individual pellets were resuspended in Tris-HCl (50 mM, pH 8.0), and treated with 1 mL of lysozyme (100 mg/mL). The sonication process was carried out for 3.5 min with 1 minute pulses at 20 kHz and an amplitude of 40 (Misonix Ultrasonic Processor, Qsonica). Samples were centrifuged, and purified by immobilized metal affinity chromatography (IMAC) using TALON® Superflow Resin (GE Healthcare). VHH protein concentration was determined by the Bradford method.

### Western blot analysis

For the Western blot (WB) analysis, 10 μg of BthTX-I and BthTX-II were reduced, electrophoresed on a 15% SDS-PAGE, and transferred to a nitrocellulose membrane (Amersham Life Science). Reactive sites were blocked with 5% skimmed milk in TBS buffer (TBSM) at 4°C overnight. After washing with TBS/0.1% Tween 20 (TBST), the strips were incubated with 0.2 mg/mL of each anti-BthTX VHH (KF498607, KF498608, KC329715 and KC329718) overnight. Strips were washed with TBST and incubated with anti-His antibody (GE Healthcare) (1:3000 in 5% TBSM) for 16 h. After washing, the samples were incubated with HRP-conjugated anti-mouse IgG produced in goat (1:3000 in 5% TBSM). Strips were washed, and the reactive signals detected after incubation with hydrogen peroxide in diaminobenzidine (DAB) solution (SIGMA*FAST*™ DAB with Metal Enhancer Tablets, Sigma-Aldrich). Llama postimmunization serum (1:1000) was used as positive control. The negative control was performed with the llama pre-immune serum (1:1000).

### Kinetic analysis

SPR spectroscopy was performed using a Biacore T200 system (GE Healthcare). BthTX-I and BthTX-II were immobilized on carboxymethylated dextran CM5 chips by amine coupling [23]. The dextran layer of the sensor chip was activated by a 1:1 (v/v) mixture of 0.4 M EDC (1-Ethyl-3-3-dimethylaminopropyl carbodiimide) and 0.1 M NHS (N-hidroxysuccinimide) at a flow rate of 5 μL/min. BthTX-I was diluted in sodium phosphate buffer (66 mM, pH 7) and injected into a selected the flow cell for surface immobilization, while BthTX-II was diluted in sodium acetate (10 mM, pH 5.5). After that, a solution of 1 M ethanolamine hydrochloride was injected in order to block remaining reactive groups in flow cell. The control flow cell was prepared only with ethanolamine. For kinetic measurements, CM5-BthTX-I and CM5-BthTX-II were subjected to serial dilutions of VHH (442.6 to 3.458 nM), and (1757.8 to 0.429 nM), respectively, into the running buffer (0.1 M phosphate buffer, 27 mM KCl and 1.37 M NaCl, pH 7.4). The kinetic assay was performed at a flow rate of 30 μL/min at 37°C. Chip regeneration was performed with AIW solution followed by ICW solution for 30 s each [**A**—Equal volumes of oxalic acid, H_3_PO_4_, formic acid, and malonic acid, each at 0.15 M, pH 5.0; **C**—20 mM EDTA; **I**—KSCN (0.46 M), MgCl_2_ (1.83 M), urea (0.92 M), guanidine-HCl (1.83 M); **W**—deionized water] according to [24]. The binding responses were calculated by subtracting the RUs obtained from both blank control cell and running buffer injection. Kinetic analyses were performed by fitting the obtained sensograms with the 1:1 Langmuir model using the BIA-evaluation software (GE Healthcare).

### Inhibition of phospholipase activity on fluorescent lipid

PLA_2_ activity was measured using acyl-NBD PC substrate (Avanti Polar Lipids) through fluorescence intensity. The fluorescent phospholipid was reconstituted in chloroform and dried over a low flow of N_2_. The dried phospholipid (Acyl-NBD-PC-1-acyl-2-{6-[(7-nitro-2-1,3-benzoxadiazol-4-yl)amino]hexanoyl}-sn-glycero-3-phosphocholine) was dissolved in 150 mM NaCl, and a stock phospholipid solution of 1 mg/mL was obtained and used throughout the experiments [25]. Assays were performed in a final volume of 150 μL in Fluorescence Spectrometer Spectra Max M4 (Molecular Devices, Sunnyvale, CA)—Software SoftMax 6.0 at 37°C in opaque plates at excitation and emission wavelengths of 460 nm and 534 nm, respectively, at an interval of 3 s between each read, over 5 min. The standard reaction medium contained 20 mM Tris–HCl pH 7.5, 8 mM CaCl_2_ and 5 μM of phospholipid. To verify VHH’s ability to inhibit BthTX-II phospholipase activity, 0.06 μg of the toxin was pre-incubated with selected VHHs (1:5; 1:10 and 1:40 w/w) for 30 min at 37°C. As a positive control the standard reaction with only BthTX-II (0.06 μg) was used. The reaction produced 220 fluorescence units, corresponding to 100% of PLA_2_ activity. The negative control was carried out in the absence of BthTX-II.

### Cross reactivity of anti-BthTX VHHs

The cross reactivity of selected VHHs was analyzed by ELISA. For this, microplates were coated separately with 500 ng of snake venoms (Serpentário de Proteínas Bioativas, Batatais, Brazil) from twenty-two different species (*B*. *alternatus*, *B*. *atrox*, *B*. *bilineata*, *B*. *brazili*, *B*. *diporus*, *B*. *insularis*, *B*. *jararaca*, *B*. *leucurus*, *B*. *marajoensis*, *B*. *matogrossensis*, *B*. *moojeni*, *B*. *pauloensis*, *B*. *pirajai*, *B*. *urutu*, *Calloselasma rhodostoma*, *Crotalus atrox*, *Crotalus durissus cascavella*, *C*. *d*. *collilineatus*, *C*. *d*. *cumanensis*, *C*. *d*. *terrificus*, *Micrurus spixii*), as well as seven different isolated toxins (PLA_2_ from *B*. *atrox*, and PLA_2_-I, convulxin, crotamin crotapotin, crotoxin, and giroxin from *C*. *d*. *terrificus*. After adsorption of the venom/toxins, plates were incubated at 4°C overnight, and washed with PBST. Nonspecific sites were blocked with BS for 2 h, washed with PBST, and 1 μg of purified VHH (pre-incubated in BS) was added. Plates were incubated overnight, washed with PBST and the anti-His antibody (1:5.000) was added. Goat anti-mouse IgG-HRP (1:10.000) was used as secondary antibody. Reactions were revealed with TMB, and measured at 450 nm. *B*. *jararacussu* venom, BthTX-I, and BthTX-II, positive controls, were used. For the negative control, ELISA wells were not adsorbed with venoms or toxins. The assay was performed in triplicate.

### Neutralization of myotoxicity by *in vitro* pre-incubation

To evaluate the VHH neutralization effect on myotoxicity induced by *B*. *jararacussu* venom, BthTX-I and BthTX-II, animals were divided into 13 groups of 5 male mice (28–30 g). *B*. *jararacussu* venom, BthTX-I and BthTX-II were separately pre-incubated with VHH at 37°C for 1 h, according to Table 1. Then, each group received an i.m. injection of 50 μL in the gastrocnemius muscle. Positive control animals were injected with BthTX-I, BthTX-II or venom. Negative control animals received 50 μL of PBS or VHHs. After 3 h, blood was collected from the orbital plexus to determine the creatine kinase activity using diagnostic kits (BIOCLIN, Belo Horizonte, MG). Results were expressed as CK U/L. To analyze the cross neutralization, 3 other groups of mice were treated with a myotoxic PLA_2_ of *B*. *brazili* (MTX-I), in the presence or absence of VHHs.

**Table 1:** Groups of mice subjected to *in vivo* neutralization assays.

Group	Description	Administered dose	Molar ratio	Administered volume
01	PBS	-	-	50 μl
02	VHH KF498607	50 μg	-	50 μl
03	VHH KC329718	50 μg	-	50 μl
04	BthTX-I	10 μg	-	50 μl
05	BthTX-I + VHH KF498607	10 μg + 50 μg	1:5	50 μl
06	BthTX-I + VHH KC329718	10 μg + 50 μg	1:5	50 μl
07	BthTX-II	15 μg	-	50 μl
08	BthTX-II + VHH KF498607	15 μg + 75 μg	1:5	50 μl
09	BthTX-II + VHH KC329718	15 μg + 75 μg	1:5	50 μl
10	*B*. *jararacussu* venom	10 μg	-	50 μl
11	*B*. *jararacussu* venom + VHH KF498607	10 μg + 50 μg	-	50 μl
12	*B*. *jararacussu* venom + VHH KC329718	10 μg + 50 μg	-	50 μl
13	*B*. *jararacussu* venom + VHH KC329718	10 μg + 100 μg	1:10	50 μl
14	MTX-I	10 μg	-	50 μl
15	MTX-I + VHH KF498607	10 μg + 50 μg	1:5	50 μl
16	MTX-I + VHH KC329718	10 μg + 50 μg	1:5	50 μl

### Molecular docking and modeling

Homology modeling was carried out with 4 selected VHH clones. Protein Blast [26] and Protein Data Bank (PDB) were used for search and retrieval of the template structures. Sequence alignment was performed by ClustalW [27] and MODELLER v9.10 [28], which was also used for model building. A total of 1000 models were generated and the final model was selected based on the lowest DOPE scores calculated. The overall stereochemical quality of the final model for VHHs was assessed using the program PROCHECK [29]. Interactive visualization and comparative analysis of molecular structures were carried out on a Swiss-PDB viewer [30] and UCSF Chimera [31], and structure images were generated by Persistence of Vision Raytracer (POV-ray) 3.62 (http://www.povray.org). The ClusPro2.0 server (http://ClusPro.bu.edu/) [32] was used to predict the possible interactions between all VHH structures and BthTX-I (PDB accession code: 3HZD:A) [33] and BthTX-II (PDB accession code: 3JR8:A) [34]. The antibody mode was used with the non-CDR regions masked automatically [35]. VHHs were submitted as the receptor and the toxins as the ligand. The software identified the 1000 best scoring solutions and then clustered them according to RMSD considerations. Subsequently, the lowest ClusPro score, which represents the greatest probability of antigen-antibody interaction, was selected [36].

## Results

### *L*. *glama* immune response and selection of anti-BthTX VHHs

**Fig 1:**
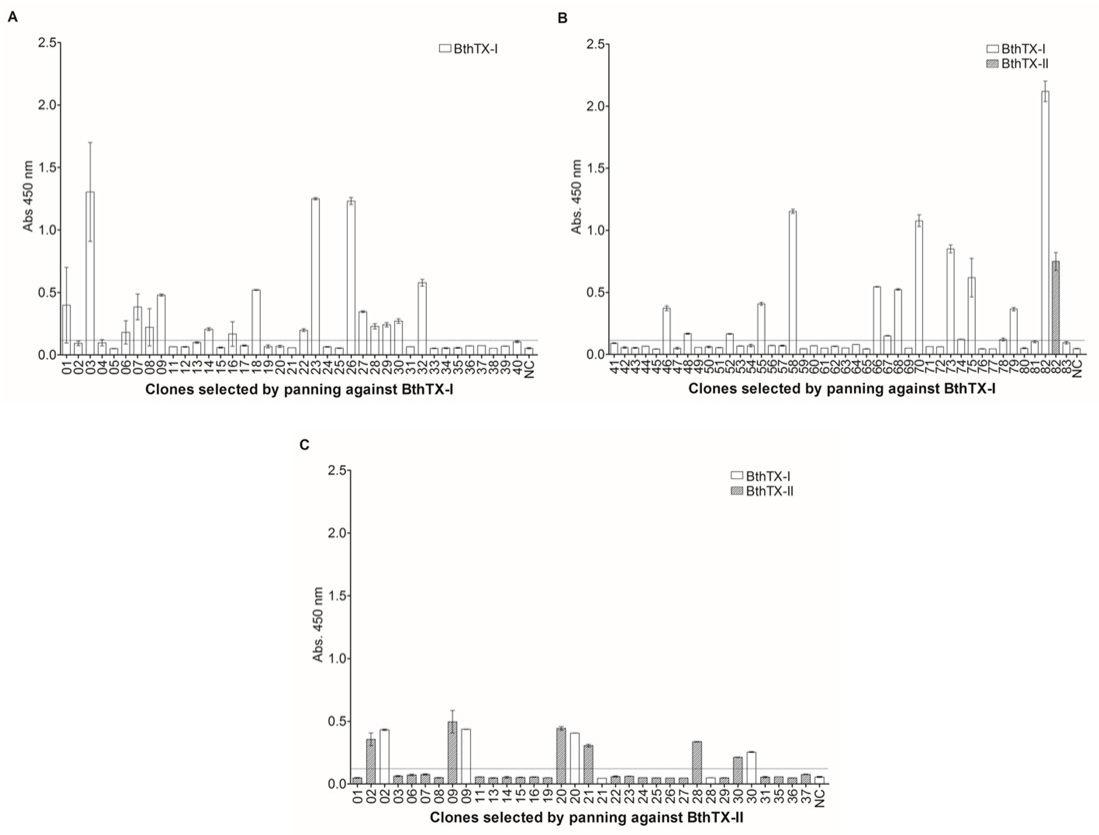
Selection of anti-BthTX-I and anti-BthTX-II VHHs. VHHs from the recombinant phage displayed library were submitted separately against immobilized BthTX-I or BthTX-II. After one round of panning, 82 and 28 clones selected for BthTX-I and BthTX-II, respectively, presented VHHs. ELISA assays were performed to verify clone reactivity. Twenty-eight (28) and six (06) clones recognized BthTX-I and BthTX-II. (A) Clonal reactivity of VHHs (1–40) selected against BthTX-I, NC–negative control; (B) Clonal reactivity of VHHs (41–83) selected against BthTX-I, Cross-reactivity of VHH 82 with BthTX-II, NC–negative control; (C) Clonal reactivity of VHHs (1–37) selected against BthTX-II, Cross-reactivity of 02, 09, 20, 21, 28 and 30 VHHs with BthTX-I, NC–negative control. All clones that showed an absorbance value (OD 450nm) higher than the stipulated cut-off point (2 mean OD from negative samples plus 2 standard deviations) were considered positive. All measurements were performed in triplicate. The negative control was performed using the llama pre-immune serum. Error bars represent standard deviation.

The llama humoral response was satisfactory, verified by an increase in the ability of postimmunization sera to bind to the antigens in the immunoenzymatic assays (data not shown). The final antiserum titre (Day 87) was determined as 1x10^5^ for BthTX-I and BthTX-II. The Phage Display method was employed to select specific VHHs for both toxins. Prior to construction of an immune VHH library with 4 x 10^12^ clones, about 5 x 10^6^ lymphocytes from immunized *L*. *glama* were isolated. The co-infection of VHH-pHEN-1-6xHis transfected TG1 *E*. *coli* strain with the M13KO7 helper phage was performed in the phage rescuing strategy, a total of 8 x 10^6^ phage particles were obtained. To carry out the selection of anti-BthTX-I and BthTX-II VHHs, the phage displayed VHH library was submitted separately for immobilized BthTX-I or BthTX-II. After 1 round of panning against BthTX-I, 83 clones were selected. Of these, 82 clones presented the expected size for VHH by PCR colony. After 2 rounds against BthTX-II, 40 clones were grown on plates, and 28 were positive for VHHs. Once the presence of VHH in the colonies was confirmed, ELISA assays were performed to verify clone reactivity. Twenty-eight and six clones recognized BthTX-I and BthTX-II, respectively (Fig 1). It is important to note that 4 clones selected for BthTX-II were also able to recognize BthTX-I. However only 1 clone, selected for BthTX-I, showed cross-reactivity with BthTX-II. All clones that showed an absorbance value higher than the stipulated cut-off point (2 mean OD from negative samples plus 2 standard deviations) were considered positive.

### Sequence analysis of anti-BthTX VHHs

**Fig 2:**
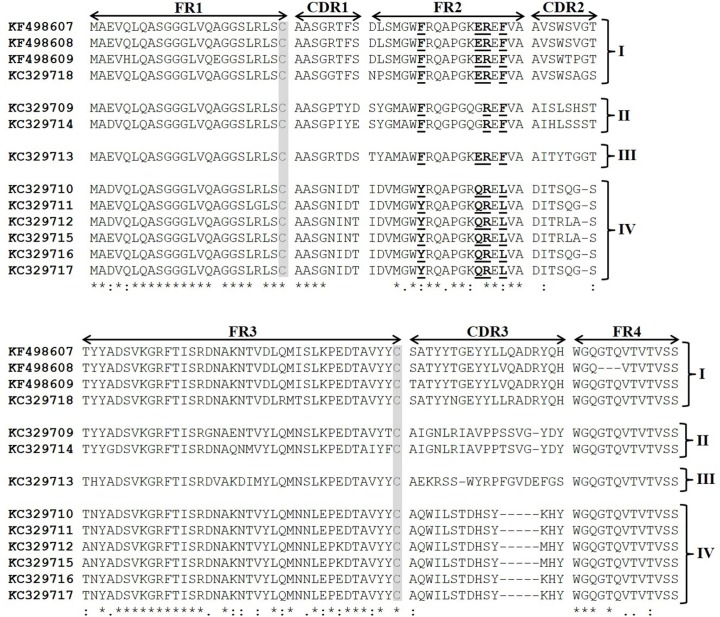
Amino acid sequence alignment of anti-BthTX-I and BthTX-II VHHs. The clones were clustered in four groups based on sequence homologies. The framework regions (FR) as well as the complementarity determining regions (CDR) are indicated with arrows; two conserved cysteines are shaded; VHH hallmark substitutions in FR2 and FR4 are bolded and underlined. Cluster I (KF498607, KF498608, KF498609, and KC329718), Cluster II (KC329709, KC329714), Cluster III (KC329713), and Cluster IV (KC329710, KC329711, KC329712, KC329715, KC329716, KC329717). The colon (:) represents highly conserved amino acids; the asterisk (*) represents identical amino acid residues; the period (.) means somewhat similar but different amino acids and blank represents dissimilar amino acids or gaps.

After sequencing 34 ELISA positive clones, a multiple sequence alignment revealed 10 sequence profiles for BthTX-I, and 3 sequence profiles for BthTX-II, which were deposited in the GenBank database under accession numbers: KC329709 (clone 9), KC329710 (clone 23), KC329711 (clone 32), KC329712 (clone 48), KC329713 (clone 58), KC329714 (clone 66), KC329715 (clone 67), KC329716 (clone 68), KC329717 (clone 75), KC329718 (clone 82), KF498607 (clone 20), KF498608 (clone 30) and KF498609 (clone 28), respectively. Based mainly on CDR homology and CDR3 length, 4 clusters of anti-BthTX VHHs (I-IV) were identified (Fig 2). The unique clone selected against BthTX-I (KC329718) that recognized BthTX-II showed a high similarity with members selected against BthTX-II. Cluster I is characterized by clones (KF498607, KF498608, KF498609, and KC329718) that presented a CDR3 length of 19 amino acid residues, with the consensus motif (S/T)ATYY(T/N)GEYYL(L/V)(Q/R)ADRYQH. Additionaly, the 4 sequences showed well-conserved CDR1 and CDR2 (consensus motif: AASG(R/G)TFS, and AVSW(S/T)(V/P/A)G(T/S), respectively). Clones that constitute Cluster II (KC329709, KC329714) and III (KC329713) also showed a long CDR3, with 18 amino acid residues. In contrast, Cluster IV, represented by 6 members (KC329710, KC329711, KC329712, KC329715, KC329716, KC329717), possess a shorter CDR3 region with a conserved motif of 14 amino acid residues (AQWILSTDHSYKHY). Moreover, CDR1 and CDR2 share highly conserved consensus sequence motifs: AASGNI(D/N)T and DIT(S/R)(Q/L)(G/A)S, respectively. Two conserved cysteine residues that form the canonical cross-species disulfide bond between FR1 and FR3, as well as the established hallmark amino acids in FR2 (Y/F37, E/Q44, R45, L/F47) that differentiate VHHs from conventional VHs were identified in the sequences. Only members of Cluster II did not present all 4 amino acid substitutions in FR2. Due to the high similarity observed between clones of Cluster I, and the great ability to recognize both proteins by ELISA, two clones KF498607 and KF498608, as well as the clone KC329718, selected during the panning for BthTX-I, were selected for futher experiments. Additionally, clone KC329715 belonging to Cluster IV, able to recognize only BthTX-I, was also investigated. After expressing VHHs (KF498607, KF498608, KC329718, KC329715) in the E. coli HB2151 strain, clones were purified by IMAC. Depending on the VHH clone, about 1 to 6 mg of recombinant protein per liter was obtained in culture flasks.

### Western blot and interaction analysis by SPR

**Fig 3:**
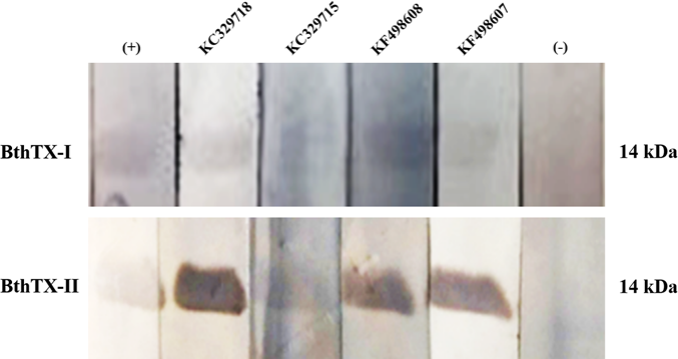
Western blot of purified VHHs against BthTX-I. 15% SDS-PAGE was carried out to resolve BthTX-I and BthTX-II under reducing conditions. After electrophoresis, BthTX-I and BthTX-II were electrotransferred to a nitrocellulose membrane and probed with selected VHHs (KF498607, KF498608, KC329715, and KC329718). Samples were incubated with mouse anti-His antibody followed by HRP-conjugated anti-mouse IgG produced in goat. Reactive signals were detected by DAB staining in the presence of hydrogen peroxide. *Lama glama* pre-immune serum was used as negative control (-), and *Lama glama* immune serum, as positive control (+).

Clones KF498607, KF498608, and KC329718 were able to bind to BthTX-I and BthTX-II, used for VHH library construction. On the other hand, KC329715 showed no or weak interaction with the toxins. Pre-immune serum was not capable of recognizing the toxins in WB (Fig 3).

**Fig 4:**
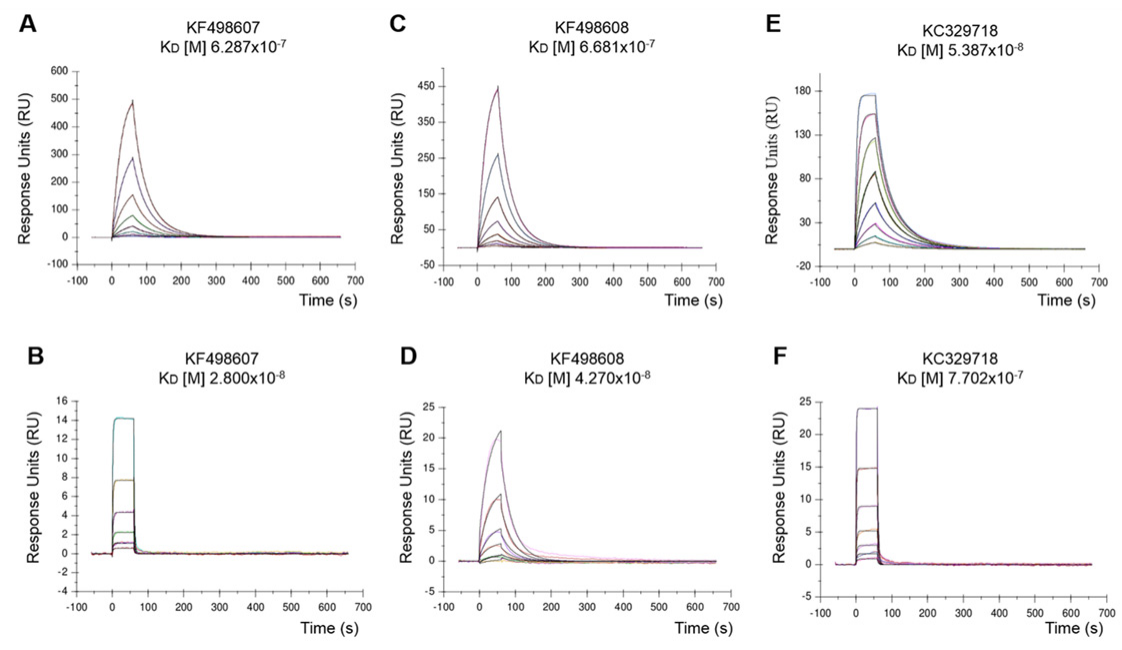
VHHs affinity to BthTX-I and BthTX-II. Representative sensorgrams of the interaction were measured in a Biacore T200 system (GE Healtcare). Purified VHHs (KF498607, KF498608 and KC329718) at concentrations of 6.25 to 0.049 μg/mL were flowed over a BthTX-I-coated CM5 chip and 25 to 0.006 μg/mL were used for immobilized BthTX-II. (A) KF498607 x BthTX-I; (B) KF498607 x BthTX-II; (C) KF498608 x BthTX-I; (D) KF498608 x BthTX-II; (E) KC329718 x BthTX-I; (F) KC329718 x BthTX-II. Assays were injected in a flow rate of 30 μl/min at 37°C. RU indicates resonance units.

The affinity between CM5-BthTX-I and CM5-BthTX-II against selected purified VHHs (KC329718, KF498607, and KF498608) measured by SPR showed binding and dissociation sensograms, with which it possible to obtain the kinetic parameters using the 1:1 Langmuir model with highly reliable fit. This was evidenced by the low Chi^2^ values obtained (< 1RU) (Table 2). All binding constants obtained were in the nanomolar range (10^−7^ to 10^−9^). According to the kinetic model, KC329718 showed the highest affinity to BthTX-I, which has a K_D_ value (53.9 nM) eleven times lower than that obtained for KF498607 (628.7 nM) and KF498608 (668.1 nM). On the other hand, KF498607 VHH showed higher affinity to BthTX-II (K_D_ of 28.0 nM) than observed for KC329718 (770.2 nM) and KF498608 (42.7 nM) (Fig 4/Table 2).

**Table 2:** Interaction analysis by SPR.

Immobilized antigen	VHH	k_on_ [1/Ms]	k_off_ [1/s]	R_max_ [RU]	K_D_ [M]	Chi2 [RU]
**BthTX-I**	KC329718	6.417x10^+5^	0.0346	185.0	5.387x10^-8^	0.709
	KF498607	2.574x10^+5^	0.1618	1,188	6.287x10^-7^	0.874
	KF498608	1.786x10^+5^	0.1193	1,110	6.681x10^-7^	0.697
**BthTX-II**	KC329718	3.347x10^+5^	0.2578	18.4	7.702x10^-7^	0.024
	KF498607	1.839x10^+5^	0.5157	60.6	2.800x10^-8^	0.014
	KF498608	7068x10^+5^	0.0302	125.7	4.270x10^-8^	0.101

Binding kinetics parameters were measured according to SPR sensorgrams shown in Fig 4 using the 1:1 Langmuir model.

### Inhibition of phospholipase activity by VHHs

**Fig 5:**
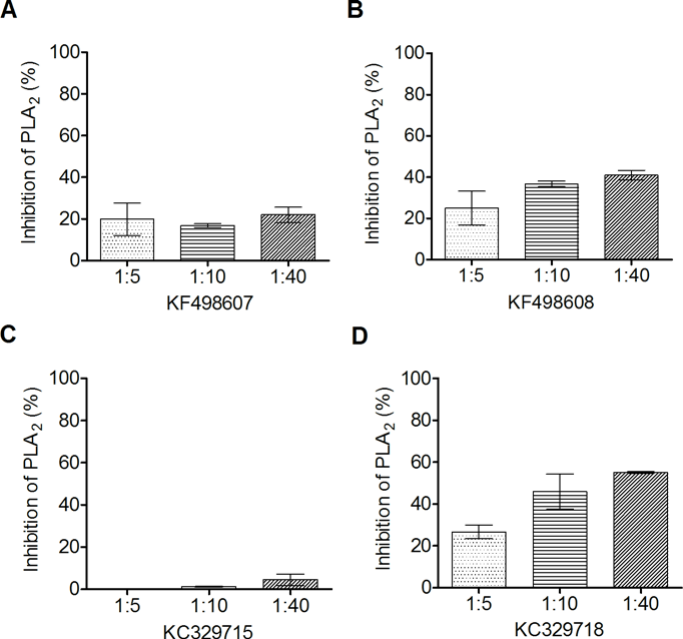
*In vitro* inhibition of phospholipase activity. The quantification of phospholipase activity inhibition by selected VHHs was assayed using synthetic fluorescent phospholipid. To verify the ability of VHHs to inhibit the phospholipase activity of BthTX-II, the toxin was pre-incubated with selected VHHs for 30 minutes at 37°C in different proportions (1:5; 1:10 and 1:40 w/w). Inhibition of phospholipase activity by (A) KF498607; (B) KF498608; (C) KF329715; (D) KC329718. BthTX-II activity on the phospholipid, in the absence of VHH, was used as a positive control, and considered as having 100% activity. The negative controls were carried out using medium reaction without BthTX-II. All measurements were performed in duplicate. Error bars represent standard deviation.

The ability of phospholipase inhibition by VHHs was assayed using synthetic fluorescent phospholipid. As shown in Fig 5, all clones presented some degree of inhibition, except the clone KC329715. The best result was obtained by KC329718, which demonstrated an inhibition of up to 60% at a ratio of 1:40 (BthTX-II:VHH).

### Cross reactivity of anti-BthTX VHHs and myotoxicity neutralization

**Fig 6:**
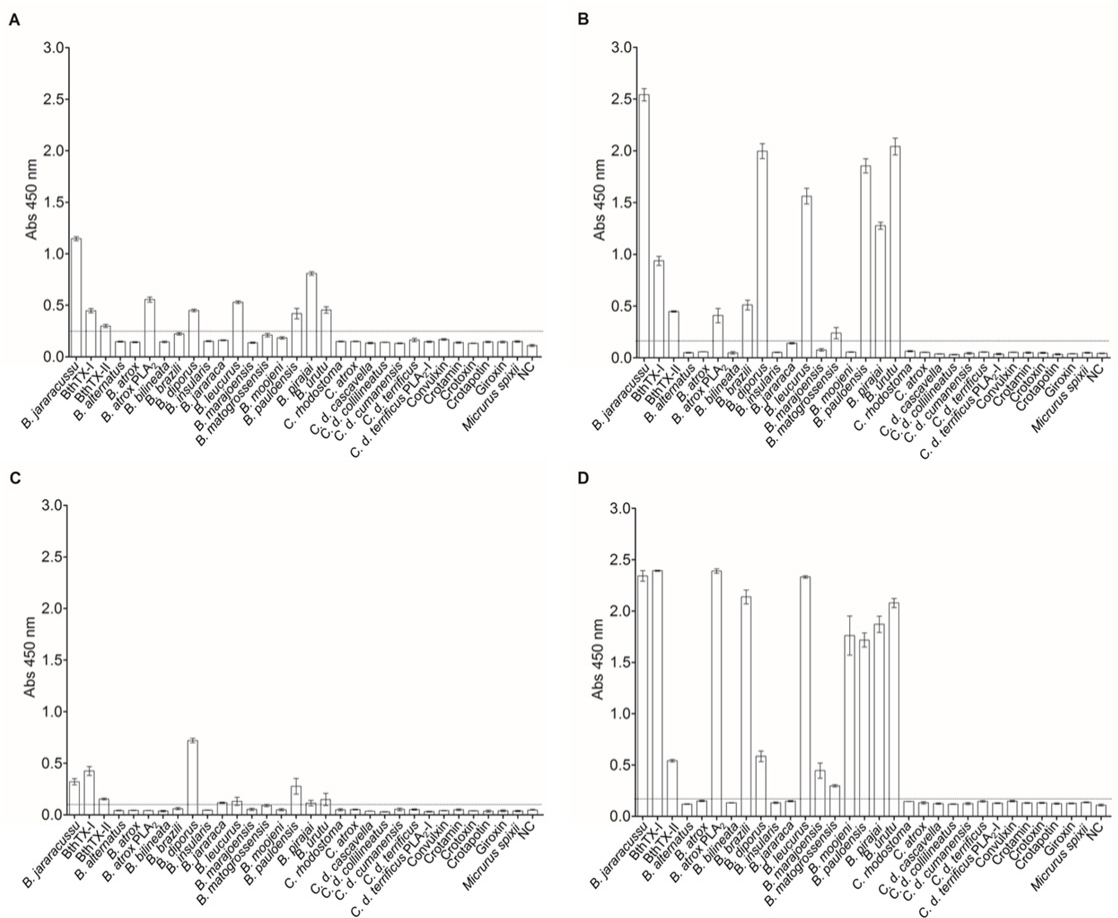
Cross-reactivity of anti-BthTX-I and BthTX-II VHHs. *In vitro* reactivity showing different levels of interaction of selected VHHs (A) KF498607; (B) KF498608; (C) KF329715; (D) KC329718 with a variety of snake venoms (*Bothrops alternatus*, *Bothrops atrox*, *Bothrops bilineata*, *Bothrops brazili*, *Bothrops diporus*, *Bothrops insularis*, *Bothrops jararaca*, *Bothrops leucurus*, *Bothrops marajoensis*, *Bothrops matogrossensis*, *Bothrops moojeni*, *Bothrops pauloensis*, *Bothrops pirajai*, *Bothrops urutu*, *Calloselasma rhodostoma*, *Crotalus atrox*, *Crotalus durissus cascavella*, *Crotalus durissus collilineatus*, *Crotalus durissus cumanensis*, *Crotalus durissus terrificus*, *Micrurus spixii*), and isolated toxins (PLA_2_ from *Bothrops atrox*, and PLA_2_-I, convulxin, crotamin crotapotin, crotoxin, and giroxin from *Crotalus durissus terrificus*). After being coated on the wells, venoms and toxins were probed with selected VHHs. Samples were incubated with mouse anti-His antibody and the reactive signals were detected after incubation with HRP-conjugated anti-mouse IgG produced in goat and TMB. All clones that showed an absorbance value (OD 450nm) higher than the stipulated cut-off point (2 mean OD from negative samples plus 2 standard deviations) were considered positive. The dashed lines represent the cut off. All measurements were performed in triplicate. For the negative control (NC), wells were not coated with venoms or toxins. Error bars represent standard deviation.

**Fig 7:**
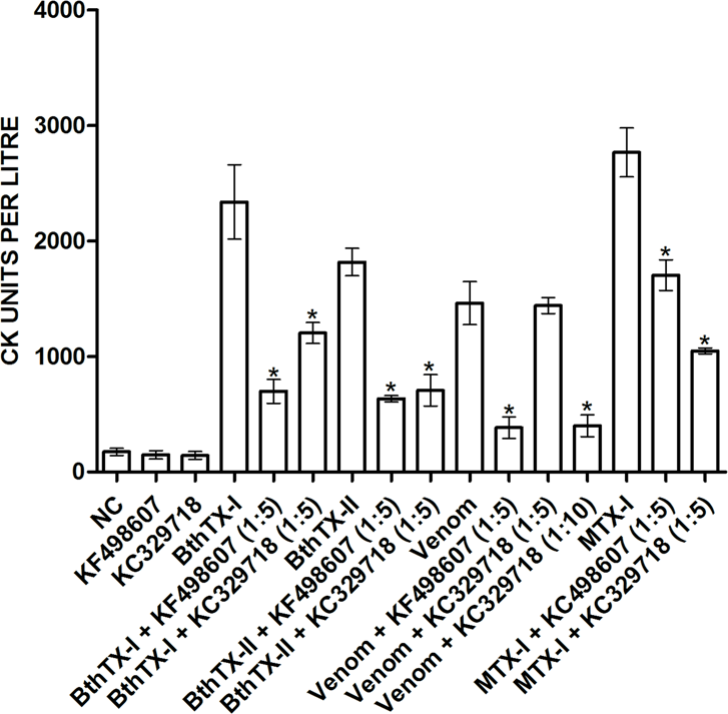
*In vivo* neutralization of *B*. *jararacussu* venom and PLA_2_-induced myotoxicity by VHHs. Plasma creatine kinase (CK) levels were measured at 340 nm to determine VHH ability to inhibit the myotoxicity caused by *B*. *jararacussu* venom or PLA_2_s, or by a related toxin, MTX-I, a PLA_2_ isolated from *B*. *brazili*. For this, groups of 5 animals were injected with PBS, VHH KF498607, VHH KC329718, BthTX-I, BthTX-I + VHH KF498607, BthTX-I + VHH KC329718, BthTX-II, BthTX-II + VHH KF498607, BthTX-II + VHH KC329718, *B*. *jararacussu* venom, *B*. *jararacussu* venom + VHH KF498607, *B*. *jararacussu* venom + VHH KC329718, MTX-I, MTX-I + VHH KC329718, MTX-I + VHH KC329718. Before administration to mice, venom or PLA_2_s and antibody preparations were pre-incubated at 37°C for 1 h, in a proportion 1:5 or 1:10 (w/w). The negative control was performed with PBS or VHHs, and as a positive control, animals were injected with *B*. *jararacussu* venom, BthTX-I, BthTX-II or MTX-I without the addition of VHH. Bonferroni’s test was used for significance analysis. (*) P <0.05. Error bars represent standard deviation.

KF498607, KF498608, KC329715 and KC329718 selected for cross reacivity showed interactions, at different levels, with the majority of venoms and PLA_2_s from different species of the *Bothrops* genus. On the other hand, the clones presented no reaction with venoms or toxins isolated from other members of the Viperidae or Elapidae families, indicating genus specificity (Fig 6). Myotoxicity induced by *B*. *jararacussu* venom, BthTX-I and BthTX-II was determined by CK activity. In a proportion of 1:5 (w/w), 10 μg BthTX-I or venom to 50 μg VHH, and 15 μg BthTX-II to 75 μg VHH, KF498607 was capable of inhibiting about 75% of the myotoxicity induced by BthTX-I, BthTX-II and venom. In the same proportion, KC329718 inhibited about 48% and 56% of BthTX-I and BthTX-II-induced myotoxicity, respectively, but showed no significant venom inhibition. However, when the assay was performed increasing the proportion to 1:10 (w/w) of venom:VHH, KC329718 presented about 73% of myotoxicity inhibition. Furthermore, when the capacity of both clones to inhibit MTX-I was analyzed, VHHs were able to neutralize around 50–55% of toxin activity (Fig 7).

### Modelling and interface binding of the VHHs and BthTX-I and BthTX-II

**Fig 8:**
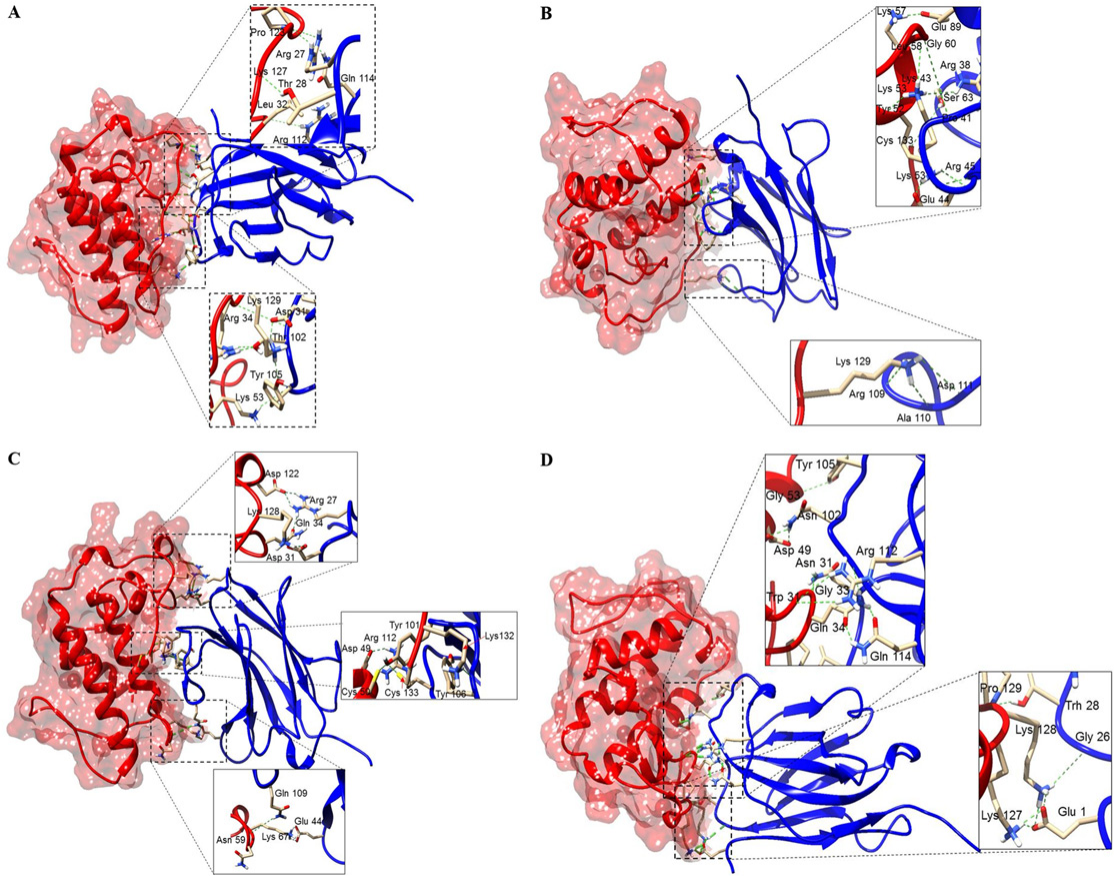
Docking results showing binding sites of VHHs on surfaces of PLA_2_s. (A) KF498607 (blue ribbon) and BthTX-I (red surface); (B) KC329718 (blue ribbon) and BthTX-I (red surface); (C) KF498607 (blue ribbon) and BthTX-II (orange surface); (D) KC329718 (blue ribbon) and BthTX-II (orange surface). The interaction sites were enlarged to show hydrogen bonds formed between the amino acid residues. Both VHHs covered the enzymatic groove surface areas of the modeled BthTX-II and inserted the CDR3 into the catalytic cleft, as well as being able to interact with amino acid residues of the PLA_2_ C-termini.

A BLAST search revealed 3 putative templates of high-level similarity with the target sequences (KF498607, KF498608, KC329715 and KC329718), as shown in Table 3. The percentage of residues lying in the favored regions of a Ramachandran plot [37] is one of the best guides to checking the stereochemical quality of a protein model. Based on the assumption that a good model should have more than 90% of the amino acid residues in the allowed regions [29], the Ramachandran plot analysis for all VHH structures indicated more than 90.04% of amino acid residues in favorable regions. The model quality was also assessed by comparing the predicted structure with the template via superimposition and atoms RMS deviation (RMSD) assessment. The RMSD of *Cα* trace between all homology structures and templates is less than 1.00 Å, supporting the idea that the generated models are reasonably good and quite similar to the templates (Table 3). The best dimeric interactions between VHHs (KF498607 and KC329718) and BthTX-I and BthTX-II, presenting the lowest ClusPro scores, are represented in Fig 8. Table 4 shows the hydrogen bonds formed between VHHs and toxins in the final docking model for KF498607 and KC329718. While results for KF498608 were quite similar to KF498607, the KC329715 VHH showed no good interaction with BthTX-I and BthTX-II.

**Table 3:** PDB code, resolution, references, percent similarity and RMSD of templates for modeled VHHs.

Target (VHH clone)	Template (PDB:chain)	% Identity	Resolution (Å)	RMSD (Å)	Reference Template
KF498607	4KRP:B	72.73	2.82	0.38	Schmitz, K.R., 2013
KF498608	4DKA:A	72.87	1.97	0.52	Park, Y.J., 2012
KC329715	3EZJ:B	70.97	2.80	0.45	Korotkov, K.V., 2009
KC329718	4KRP:B	73.85	2.82	0.33	Schmitz, K.R., 2013

**Table 4:** Molecular interactions between the amino acid residues of VHHs and PLA_2_s.

Clone	VHH	Amino acid residue		Toxin Domain	Antigen
	Domain	Antibody	Antigen		
**KF498607**	CDR1	Arg27	Pro123	C-terminal	**BthTX-I**
	CDR3	Gln114	Pro123	C-terminal	
	CDR1	Thr28	Lys127	C-terminal	
	CDR3	Arg112	Leu32	Ca^++^-binding site	
	FR2	Asp31	Lys129	C-terminal	
	CDR3	Thr102	Arg34	Ca^++^-binding site	
	CDR3	Tyr105	Lys53	α-2 helix	
**KC329718**	FR3	Glu89	Lys57	α-2 helix	**BthTX-I**
	FR2	Arg38	Lys53	α-2 helix	
	FR2	Lys43	Leu58	α-2 helix	
	FR2	Lys43	Tyr52	α-2 helix	
	FR3	Ser63	Cys133	C-terminal	
	FR2	Pro41	Gly60	α-2 helix	
	FR2	Arg45	Lys53	α-2 helix	
	FR2	Glu44	Lys53	α-2 helix	
	CDR3	Asp111	Lys129	C-terminal	
	CDR3	Arg109	Lys129	C-terminal	
	CDR3	Ala110	Lys129	C-terminal	
**KF498607**	CDR1	Arg27	Asp122	C-terminal	**BthTX-II**
	CDR1	Arg27	Gln34	Ca^++^-binding site	
	FR2	Asp31	Lys128	C-terminal	
	CDR3	Tyr101	Cys133	C-terminal	
	CDR3	Tyr101	Cys133	C-terminal	
	CDR3	Tyr106	Lys132	C-terminal	
	CDR3	Arg112	Asp49	Catalytic site	
	CDR3	Gln109	Asn59	α-2 helix	
	FR2	Glu44	Lys67	α-2 helix	
**KC329718**	CDR3	Tyr105	Gly53	α-2 helix	**BthTX-II**
	CDR3	Asn102	Asp49	Catalytic site	
	CDR3	Arg112	Trp31	Catalytic site	
	FR2	Asn31	Gly33	Ca^++^-binding site	
	CDR3	Gln114	Gln34	Ca^++^-binding site	
	CDR1	Thr28	Pro129	C-terminal	
	CDR1	Gly26	Lys128	C-terminal	
	FR1	Glu1	Lys128	C-terminal	
	FR1	Glu1	Lys127	C-terminal	

## Discussion

Over the years, antivenom production has taken advantage of technological improvements in antigen preparation, immunization strategies, antibody fragmentation, as well as in the purification and preservation of antivenoms. These approaches have ensured advances in terms of activity, stability and antivenom safety, especially with regard to the hypersensitivity reactions and to the risk of zoonosis transmission [38,39]. Although antivenoms prevent or reverse most of the effects observed in snakebite victims, minimizing the mortality and physical sequelae occasioned by the envenoming, the inhibition of local tissue damage remains the major challenge for antivenom therapy [39].

Through advances in antibody engineering, monoclonal antibody fragments, which are able to quickly reach dense tissues and stop the progress of local damages, have been selected against venom relevant epitopes [10,14]. In this way, we selected VHHs against BthTX-I and BthTX-II from *B*. *jararacussu* venom.

Considering that PLA_2_s, frequently abundant in snake venoms from the Viperidae family [40], contribute substantially to local myotoxicity, anti-BthTX VHHs could limit the tissue necrosis and sequelae induced by bothropic envenoming.

To identify anti-BthTX VHHs, we produced an immune VHH library after the immunization of a *Lama glama* with purified BthTX-I and BthTX-II. Following a protocol based on “low dose, low volume, multi-site immunization” described by [41], with modifications in terms of adjuvant usage, injection volumes, and intervals between the immunizations, the animal generated a satisfatory immune response to both toxins. The high antiserum titre exhibited for BthTX-I and BthTX-II at the end of the immunization schedule is comparable to studies that used snake venoms to immunize camelids [14]. Furthermore, the animal did not present visible signs of local myotoxicity at the injections sites, despite simultaneous administration of both toxins.

With >10^7^ individual clones, the generated library seemed to ensure a high diversity of the llama VHH gene repertoire [42]. Besides that, after the construction of a phage-displayed VHH library and 1 round of biopanning for BthTX-I and 2 rounds for BthTX-II, VHH inserts were detected in 99% and 70% of the selected phagemids, respectively. Inspired by the work of [43], in which with a unique round of panning, VHHs able to inhibit the enzymatic activity of the Botulinum toxin in a nanomolar range were selected, the individual clones were analyzed by ELISA and the sequencing of positive clones were performed.

Despite a high similarity (about 70%) between BthTX-I and BthTX-II [20], indirect ELISA revealed that only 1 clone was able to interact with BthTX-II. On the other hand, 4 clones selected against BthTX-II presented cross reactivity with BthTX-I, indicating that these VHHs would interact with similar epitopes of both toxins and may even originate from the same B-cell lineages.

Sequence analysis showed that the clones selected against BthTX-I and BthTX-II presented the VHH hallmarks in 13 different profiles. The amino acid substitutions reshape the side that interacts with the VL domain, preventing the binding to the light chain. Besides that, these substitutions promote an increase in VHH solubility and thermostability [44,45]. According to [14], VHHs based on the high CDR sequence identity and CDR3 length were grouped. All 4 clusters presented a CDR3 containing between 14 and 19 amino acid residues, which could enable its insertion into sites not usually accessible to conventional antibodies [16]. The unique clone (KC329718) selected against BthTX-I that recognized both proteins showed about 90% identity with clones selected against BthTX-II, reinforcing the idea that these VHHs interact with common antigenic sites of the proteins.

In order to verify VHH ability to specifically recognize the PLA_2_, Western Blot analysis was performed. While *L*. *glama* post-immune serum and KF498607, KF498608, KC329718 VHHs were able to detect the reduced proteins in different degrees of reactivity, KC329715 VHH presented weak or no interaction with BthTX-I and BthTX-II, respectively. This is probably due to the difference in affinity between the clones.

Therefore, an SPR assay was used to evaluate the kinetic interactions between the selected VHHs and BthTX-I and BthTX-II. When compared with other anti-BthTX VHHs, the KC329718 VHH showed the highest affinity for BthTX–I (K_D_ 53.8 nM), similar to K_D_ values ​obtained by VHH selected against AahI, a neurotoxin from *Androctonus australis hector* scorpion, that was able to neutralize the toxin activity in 100% of treated swiss mice [46].

Additionally, *in vitro* assays, performed to investigate the ability of selected VHHs to inhibit the phospholipase activity of BthTX-II, demonstrated that 3 clones were capable of reducing this activity by up to 60%. Despite molar excess of VHHs over toxin does not improve PLA_2_ inhibition significantly, the data suggest that these VHHs could interact with amino acid residues of the toxin enzymatic cleft, as described by [47].

Moreover, the clones presented cross reactivity with PLA_2_s or venoms from different *Bothrops* species, indicating specificity and that VHHs react with similar antigenic sites of orthologous proteins, important features for snakebite diagnostic or serumtherapy [48,49]. It is important to note that in all *in vitro* experiments, the KC329715 VHH demonstrated lower or no reactivity against the tested venoms and toxins. This may possibily be due to smaller CDR2 and CDR3, observed among the members of cluster IV (Fig 2).

In *in vivo* assays carried out with KC329718 and KF498607 VHHs clearly demonstrated a significant decrease in mice serum CK levels, inoculated with BthTX-I, BthTX-II or *B*. *jararacussu* venom. A higher concentration of KC329718 enhanced its inhibitory effect upon BthTX-I, BthTX-II and venom-induced myotoxicity. Although 5-fold molar excess over toxins, selected VHHs were not able to abolish the myotoxicity induced by the PLA_2_s and venom, as observed in the neutralization of *B*. *asper* myotoxins using murine monoclonal antibody at a 1:1 molar ratio [50]. Despite having a 12-fold greater affinity for BthTX-I and showing higher ability to inhibit *in vitro* PLA_2_ activity of BthTX-II, the clone KC329718 seems to present lower ability to neutralize *in vivo* myotoxicity induced by *B*.*jararacussu* venom or BthTX-I. This probably occurs due to the differences between epitope specificity indicated in the molecular docking (Table 4).

In addition, the *in vivo* results confirmed the cross-reactivity of the ELISA tests, when both clones were able to inhibit MTX-I activity. Given that several studies show antivenom cross neutralization activity on specific venom toxicities of heterologous species in animal studies [49,51], our data points to the VHH’s relevance in the neutralization and cross neutralization of local damages caused by *Bothrops* venom. However, both specificity and high affinity are critical parameters of antibodies for therapeutic utility. Thus, protein engineering and mutagenesis strategies could be used for the enhancement of VHH affinity.

Previous studies indicate that myotoxic PLA_2_s target the sarcolemma as their primary site of action, which leads to a prominent influx of calcium ions and an efflux of potassium and ATP, besides other intracellular markers, inducing an acute degeneration of skeletal muscle fibers [52–54]. To trigger these events, these PLA_2_s can act by two mechanisms: i. hydrolysis of glycerophospholipids at the sn-2 position of the glycerol backbone, releasing fatty acids and lysophospholipids, a mechanism depending on Ca^++^ [47]; ii. direct cytolysis, in cases of proteins that show either no, or low catalytic activity [55]. This mechanism involves an allosteric transition and two independent interaction sites with the target membrane, i.e., cationic membrane docking site (MDoS), and hydrophobic membrane-disruption site (MDiS) [53].

Molecular docking suggested that KC329718 and KF498607 VHHs, members of Cluster I, bound to amino acid residues of the toxin C-termini, which may constitute putative MDoS of BthTX-I and BthTX-II, as suggested by [53], avoiding the protein-membrane docking. When compared with further clusters, the presence of conserved amino acid residues into VHH CDR3 (Ala110, Asp111, Arg112, Gln114) could significantly influence the interaction between VHHs and both toxins (Fig 2/Table 4). Besides that, both ligands seem to bind the Asp49 residue of BthTX-II’s enzymatic cleft, inhibiting the binding of Ca^++^, essential to its catalytic activity [47]. The formation of a hydrogen bond between Arg112 of KC329718 and Trp31 of BthTX-II may contribute to the decrease of the toxin’s phospholipase activity, as shown in Fig 5, since this amino acid residue also participates in the catalytic reaction [56]. Co-crystallization assays are being performed in order to confirm the results and to understand the myotoxic mechanism of both proteins.

Taken together, selected VHHs are able to recognize specifically BthTX-I and BthTX-II as well as to decrease the myotoxicity of *Bothrops jararacussu* venom. This inhibition is directly related to VHH insertion in the catalytic cleft and interaction with the C-termini of these phospholipases, essential for myotoxic activity. Therefore, these clones could represent a convenient tool for investigating action mechanisms of toxins, bioprospection, the development of alternative methods to snakebite diagnosis, and even as therapeutic agents to overcome the challenges of the current serumtherapy.
